# Increasing Physical Activity in Under-Resourced Communities Through School-Based, Joint-Use Agreements, Los Angeles County, 2010–2012

**DOI:** 10.5888/pcd10.120270

**Published:** 2013-05-30

**Authors:** Mariah Lafleur, Eloisa Gonzalez, Liz Schwarte, Rajni Banthia, Tony Kuo, Joanie Verderber, Paul Simon

**Affiliations:** Author Affiliations: Eloisa Gonzalez, Tony Kuo, Paul Simon, Los Angeles County Department of Public Health, Los Angeles, California; Liz Schwarte, Ad Lucem Consulting, Oakland, California; Rajni Banthia, Field Research Corporation, San Francisco, California; Joanie Verderber, Los Angeles County Office of Education, Los Angeles, California.

## Abstract

**Introduction:**

Few studies have examined how joint-use agreements between schools and communities affect use of school facilities after hours for physical activity in under-resourced communities. The objective of this study was to assess whether these agreements can increase community member use of these opened spaces outside of school hours.

**Methods:**

Trained observers conducted school site observations after joint-use agreements were implemented in 7 Los Angeles County school districts. All 7 districts had disproportionately high adult and child obesity rates, and all had executed a joint-use agreement between schools and community or government entities from January 2010 through December 2012. To assess use, we adapted the System for Observing Play and Recreation in Communities (SOPARC) instrument to record the number, demographic characteristics, and physical activity levels of community members who used the joint-use school sites. To supplement observations, we collected contextual information for each location, including the existence of physical activity programs at the site and the condition of exercise equipment.

**Results:**

We completed 172 SOPARC observations and related environmental assessments for 12 school sites. Observations made on 1,669 site users showed that most of them were Hispanic and nearly half were adults; three-quarters engaged in moderate to vigorous physical activity. Community member use of school sites was 16 times higher in joint-use schools that had physical activity programs than in schools without such programs.

**Conclusion:**

Joint-use agreements are a promising strategy for increasing moderate to vigorous physical activity among adults and children in under-resourced communities. Providing physical activity programs may substantially increase after-hours use of school facilities by community members.

## Introduction

Recognizing that environments influence physical activity levels, many community leaders, especially in low-income areas, are considering shared or joint use of school grounds and facilities to increase physical activity in the community and to improve a community’s social cohesion (1–5). A joint-use agreement employs a contract or written agreement between a school and a city or community entity outlining specific terms and conditions for shared use (6). To date, few studies have examined the use of these agreements to create physical activity opportunities in under-resourced communities (1,4,5,7).

Although many public health experts consider joint-use agreements a viable strategy for increasing physical activity, some schools remain reluctant to enter into such agreements because of concerns about liability and additional maintenance costs (6). To address these concerns, groups specializing in public health law have begun to create resources to support communities that are considering adopting joint-use agreements (6–8).

At the local level, joint-use agreements are an attractive approach for increasing physical activity among residents, especially for communities with limited parks or open spaces and high rates of obesity (9). A recent report by the Los Angeles County Department of Public Health (DPH) showed that cities in the county with the least available amount of park space experienced more economic hardship and had higher obesity rates than cities in the county with the most available amount of park space (10).

With the support of the Communities Putting Prevention to Work obesity prevention program of the Centers for Disease Control and Prevention (http://www.cdc.gov/communitiesputtingpreventiontowork/), DPH established the Joint Use Moving People to Play (JUMPP) Task Force to address the lack of physical activity opportunities by making system and environmental changes, such as joint-use agreements with schools. Our study examines the JUMPP Task Force’s work from January 2010 to December 2012. Our study’s objective was to assess whether joint-use agreements can increase community members’ use of school grounds outside of school hours.

## Methods

We used mixed methodology to evaluate community member use of school grounds following execution of JUMPP-assisted joint-use agreements in Los Angeles County from January 2010 to December 2012. Methods included cross-sectional observations of community members’ use of school facilities and environmental assessments of facilities and exercise equipment at each site. Although our evaluation team envisioned pre- and post-test design data collection (ie, onsite observations of physical activity before and after implementation of joint-use agreements), school grounds or facilities were not opened to the community outside of school hours until joint-use agreements were executed, making pre-implementation observations unnecessary. Agreements were executed throughout Los Angeles County; however, these agreements were concentrated in areas with the least amount of park space per 1,000 residents (Figure).

**Figure:**
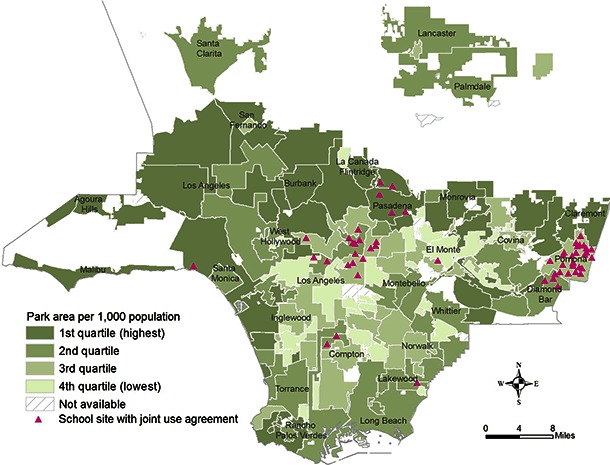
School sites with the Joint Use Moving People to Play Task Force-assisted joint use agreements, by city or community and by park areas per 1,000 residents. Map of Los Angeles County prepared by the Office of Health Assessment and Epidemiology in the Los Angeles County Department of Public Health.

### Context and setting

Los Angeles County has 9.8 million residents and a land area of 4,058 square miles. This complex geopolitical jurisdiction comprises 88 cities, 80 school districts, a large unincorporated area governed by Los Angeles County, and several multiethnic communities. Forty-seven percent of the Los Angeles County population is Hispanic (9). In March 2010, DPH launched its obesity prevention program component of Communities Putting Prevention to Work, RENEW LA County (RENEW) (11). One of RENEW’s program objectives was to implement joint-use agreements between schools and communities in under-resourced communities across the county. Guided by DPH’s JUMPP Task Force, RENEW’s joint-use program was designed to increase access to recreational space, school facilities, and physical activity programs through the adoption and implementation of joint-use agreements in 7 school districts, from December 2010 to January 2012.

Seven school districts participated in the JUMPP effort: ABC Unified School District, which comprises the cities of Hawaiian Gardens, Artesia, and Cerritos; Compton Unified School District; El Monte City School District; Los Angeles Unified School District; Mountain View School District; Pasadena Unified School District; and Pomona Unified School District. The JUMPP Task Force invited only districts with a high child obesity burden, identified by using the California Department of Education’s FITNESSGRAM physical fitness test data to determine eligibility to participate in RENEW. The FITNESSGRAM physical fitness test is a comprehensive, health-related physical fitness examination comprising a variety of physical tasks and height and weight measurements. It is aimed at assisting students in establishing lifetime habits of regular fitness activity, and all California students in grades 5, 7, and 9 are required to take the FITNESSGRAM Physical Fitness Test (12). The estimates of obesity burden were based on calculated obesity rates from FITNESSGRAM data for public school students in the 5th, 7th, and 9th grades (13). The student obesity rates for the participating school districts were in the top one-third of all public schools in the county; they exceeded the county average obesity rate of 22.9% for 2007 by as much as 7.0% to 27.0% (13). We used the Los Angeles County Health Survey to document comparable adult obesity rates for the cities and communities surrounding the participating districts (9).

### School sites that adopted and implemented joint-use agreements

JUMPP-assisted joint-use agreements established from January 2010 through December 2012 allowed both adults and children to use the school space and covered a time period of at least 2 years, beginning on the date of contract execution. Participating schools developed their own individual agreements, which were based on model language provided by ChangeLab Solutions (8). JUMPP worked to help school districts that had existing joint-use agreements that did not permit use of school facilities by the entire community to modify or expand the scope of their agreements.

In addition to opening up the school grounds for community use, 10 of the 12 agreements included an organized physical activity program for adults or children. Programs at these sites included swimming, aerobic dance classes, golf, exercises using fitness video games, tennis classes, and walking clubs. Partners such as the city and county departments of parks and recreation, the American Youth Soccer Organization, and local golf and tennis organizations conducted programs. In most cases, these partner organizations provided both funding and staffing for the programs.

Most of the agreements executed by participating school districts affected schools located in or near low-income neighborhoods, typically in places where residents had limited access to open space. The Los Angeles County Office of Education facilitated the development, adoption, and implementation of the joint-use agreements for 6 of the 7 participating school districts (Los Angeles Unified School District facilitated its own). With district administrators’ permission, the evaluation team observed use and activity levels at each of the participating school sites after each agreement had been executed. Prior to the initiation of RENEW, most joint-use agreements that were in place involved only programs for youth, and the sites were not formally open to the entire community.

### Data collection

Trained observers conducted the joint-use site observations and assessments from April 2011 through February 2012. Observers began data collection as agreements were adopted and implemented. The evaluation team adapted the System for Observing Play and Recreation in Communities (SOPARC) tool from McKenzie et al (14) as the primary study instrument for collecting data on the number, demographics, and physical activity levels of community members who used the joint-use school sites. The adapted SOPARC simplified the age group and race/ethnicity observations by documenting only whether the observed persons appeared to be aged under (child) or over (adult) 18 years and whether they appeared to be white or nonwhite. The observers were trained using the SOPARC introduction, assessment, practice, and training DVD and the SOPARC protocols, data path coding forms, and mapping strategies documents (15). Mock fieldwork–trainings were provided as needed. This training was a refresher course for most observers, who had previous experience using the SOPARC observational tool. Because of resource and time constraints, only 1 observer at a time was able to make observations at each site. As a result, the evaluation team could not carry out a meaningful assessment of inter-rater reliability.

In addition to assessing physical activity levels, trained observers collected contextual information on the setting in which the physical activity occurred, including weather and temperature and whether an organized activity or program took place during the observation. While on site, observers measured the size of each activity space and assessed the condition of available equipment and whether the spaces were conducive to physical activity. Observers defined “conducive to physical activity” as being safe for physical activity and free from standing water, excessive litter, and broken glass. The evaluation team conducted the environmental assessments to complement data from the SOPARC observations.

Observers assessed each school site on 2 separate days during times when the space was available for use. They made multiple observations each day that were evenly distributed throughout the period of time when the space was available for shared use. The number of observations conducted per day was proportional to the length of time the joint-use site was open (ie, 4 sets of observation when a space was open for 4 or more hours and 3 sets of observation when the space was open for 2 or 3 hours). Joint-use hours at the school sites ranged from early morning before school opening, afternoon and evening after school closing, and longer hours from morning to evening on weekend days.

### Data analysis

The evaluation team analyzed all data collected from the SOPARC observations and related environmental assessments by using SPSS (SPSS Version 20.0, IBM Corp, Armonk, New York).

The team generated descriptive statistics and frequencies to summarize study results. When appropriate, the team averaged multiple observations to yield summary descriptions of key outcomes. On the basis of the initial descriptive work, the team formulated an analysis plan to compare data from all 12 joint-use sites, stratifying by physical activity programs (ie, comparing the use frequencies of sites with and without physical activity programs). To provide context for the intervention’s reach, the analysis synthesized and mapped geodemographic data to describe joint-use locations in relation to park space in Los Angeles County (16). The evaluation team used ArcGIS 10.0 (ESRI, Redlands, California) to perform all mapping. DPH’s institutional review board reviewed and approved all study protocols, procedures, and materials.

## Results

The combined total amount of space available for physical activity at all 12 observed sites was 43,101 square meters; available space for each site ranged from 155 square meters to 10,787 square meters. Eleven of the 12 sites were deemed conducive to physical activity per SOPARC criteria and had equipment that was in working condition. The 1 school site deemed not conducive to physical activity had an area with excessive mud and some standing water during 1 of the days of observation. Of all spaces observed, 83% were outdoor areas and 17% were indoors; spaces were all open and accessible during the time of observation. On most days of site visits, the weather was mild; observers did not encounter rainy or excessively hot conditions.

In total, the evaluation team completed 172 SOPARC observations and environmental assessments for the 12 school sites with joint-use agreements. Observers recorded information for a total of 1,669 community members; nearly half (42.0%) were adults and almost all (95.0%) were nonwhite (Table 1). The age and race characteristics of site users (Table 2) were similar to those of the population in the surrounding communities (Table 3).

**Table 1:** Age and Race Characteristics of Adults And Children (N = 1,660)^a^ Observed in School Sites With Joint-Use Agreements, Los Angeles County, 2010–2012

Demographic Characteristic	n (%)
**Age**	
Adults	702 (42)
Children	967 (58)
**Sex**	
Men	881 (54)
Women	758 (46)
**Race**	
White	86 (5)
Nonwhite^b^	1,576 (95)

a Sum of n among age, sex and race categories varies slightly as a result of data collection methodology.

b Denotes Hispanic, Asian, and black race/ethnicity as determined by the observer.

**Table 2:** Characteristics of Users of School Sites in 7 School Districts With Joint-Use Agreements, Los Angeles County, 2010–2012

District	School	Grade Level	Enrollment	% Eligible for Free or Reduced Price Meals	Male ,%	Female, %	White, %	Nonwhite, %
Compton Unified	Carver Elementary	K–5	354	93.2	53.4	46.6	0.3	99.7
El Monte City	Columbia	K–8	907	92.5	48.7	51.3	0.8	99.2
Los Angeles Unified	School of Engineering and Technology	9–12	403	81.9	51.4	48.6	0.2	99.8
Los Angeles Unified	School of Math and Science	9–12	409	82.9	51.6	48.4	0.2	99.8
Los Angeles Unified	Helen Bernstein High School	9–12	1,475	73.6	53.8	46.2	8.3	91.7
Los Angeles Unified	Euclid Elementary	K–5	1,056	95.9	45.5	54.5	0.3	99.7
Los Angeles Unified	Miguel Contreras Learning Complex	9–12	939	86.1	48.0	52.0	0.1	99.9
Mountain View	Miramonte Elementary	K–6	563	95.8	48.8	51.2	0.9	99.1
Pomona Unified	Cortez Mathematics and Science Magnet	K–8	690	86.0	50.1	49.9	2.8	97.2
Pomona Unified	Allison Elementary	K–6	400	85.5	51.2	48.8	2.8	97.2
Pomona Unified	Palomares Academy of Health Sciences	6–8	388	95.1	52.8	47.2	3.1	96.9
Pasadena Unified	Cleveland Elementary	K–6	332	85.8	49.7	50.3	2.1	97.9
Pasadena Unified	McKinley	K–8	1,116	55.5	50.1	49.9	14.4	85.6
Los Angeles Unified	Marquez Elementary	K–5	599	11.7	54.4	45.6	74.0	26.0

**Table 3:** Characteristics of Communities Near School Sites With Joint-Use Agreements, Los Angeles County, 2010–2012 (24)

District	School	Population	Residents Below 185% Poverty Level, %*	Male, %	Female, %	White, %	Nonwhite, %
Compton Unified	Carver Elementary	37,335	47.0	47.3	52.7	0.7	99.3
El Monte City	Columbia	22,421	48.2	50.2	49.8	3.5	96.5
Los Angeles Unified	School of Engineering and Technology	21,325	52.5	67.5	32.5	14.6	85.4
Los Angeles Unified	School of Math and Science						
Los Angeles Unified	Helen Bernstein High School	46,213	53.1	51.8	48.2	30.6	69.4
Los Angeles Unified	Euclid Elementary	25,183	54.2	49.8	50.2	1.2	98.8
Los Angeles Unified	Miguel Contreras Learning Complex	50,872	65.8	53.7	46.3	9.7	90.3
Mountain View	Miramonte Elementary	25,425	50.6	50.7	49.3	2.6	97.4
Pomona Unified	Cortez Mathematics and Science Magnet	22,861	37.3	50.0	50.0	16.1	83.9
Pomona Unified	Allison Elementary	27,115	32.7	49.4	50.6	16.0	84.0
Pomona Unified	Palomares Academy of Health Sciences	4,815	38.9	48.9	51.1	11.2	88.8
Pasadena Unified	Cleveland Elementary	17,457	26.6	48.5	51.5	24.0	76.0
Pasadena Unified	McKinley	25,061	17.0	48.9	51.1	50.9	49.1
Los Angeles Unified	Marquez Elementary	9,832	6.7	47.3	52.7	85.2	14.8

Ten of the school sites had physical activity programs underway some or all of the time that their facilities were open to shared use; 2 sites did not offer any programs. Observers conducted nearly half (47.0%) of the 172 observations and environmental assessments while physical activity programs was underway at the site. The spaces across all sites were empty one-third (35.0%) of the time, but only at times when no programs were underway. The 2 sites that did not offer physical activity programs were empty more frequently than sites that offered programs. Community use was 16 times higher at joint-use sites when observations were conducted during physical activity programs than when no structured programs were underway (*P* < .001). In observations made during programs, an average of 8.4 observed adults and 11 children used the school grounds or facilities; in observations when there was no program underway, 0.3 adults and 0.9 children used the school grounds or facilities.

About two-thirds (68.0%) of all community members using school facilities were observed participating in moderate (51.0%) or vigorous (17.0%) physical activity. The remaining community members were inactive at the time of observation (32.0%). The evaluation team did not find differences in observed physical activity levels (ie, moderate vs vigorous) between users at sites with physical activity programs and users at sites without programs.

## Discussion

Our study showed that adopting joint-use agreements is feasible for schools and can successfully attract community members to use school facilities for physical activity outside of school hours. The study also showed that conducting physical activity programs for adults and children at schools increases community use of school facilities. Because most JUMPP-assisted agreements were adopted and implemented in cities or communities with disproportionately high obesity rates, this strategy could be a model of practice for similar communities. It would align with the emerging viewpoint among community leaders that joint-use agreements are worthwhile investments that can increase physical activity opportunities in under-resourced areas of a city or community at a relatively low cost (6,8).

Physical activity programming has been found in several studies to be a major factor in promoting moderate to vigorous physical activity and community use of parks and open spaces (17,18). In this study, school sites with physical activity programs attracted 16-fold more community members than school sites that had no structured programs. With guidance from the JUMPP task force and support from partnering organizations (eg, city and county parks and recreation departments, the American Youth Soccer Organization), physical activity programs can become an integral part of interventions, helping to sustain and accentuate the health and social benefits of joint-use agreements over time.

Another important finding of our study is the value of adult-inclusive programs for attracting both adults and children to school sites during joint-use hours. This finding alludes to a family unit effect, suggesting that adult users will bring their children along for the structured activity even if specific child-centered activities are not offered. Interestingly, both adults and children accessing school recreational facilities during joint-use hours engaged primarily in moderate to vigorous physical activity regardless of the presence or type of program. Among these users, Hispanics remained the largest group seen engaging in moderate to vigorous physical activity. This observation has important implications for reducing health disparities given the disproportionately high rates of obesity and weight-related chronic conditions in Hispanic populations (19,20).

Our study has several limitations. First, the observational nature of the evaluation design limited collection of individual-level data from community members, including such measures as self-reported sociodemographics, customary recreational habits, and intentions to change physical activity behaviors. This limitation, however, should not be viewed as a weakness of the observational data, because these observational data are generally regarded as more reliable than self-reported behaviors, avoiding, in many instances, social desirability and selection biases (21). Second, because of limited resources and time constraints, only 1 trained observer was dispatched to each site for the SOPARC observations. For this reason, inter-rater reliability could not be meaningfully assessed. Although this may represent a notable unknown about the adapted SOPARC tool’s internal validity, the concern is likely minimal because the tool has been previously validated by its designers (14), and the adaptations that we made did not deviate significantly from the original instrument. Third, the cross-sectional data collection approach offered no temporal information about changes in physical activity levels among community members. For example, there is no way to determine whether community members observed using the school facilities actually increased their net amount of physical activity as a result of the joint-use agreements. Users may possibly have substituted physical activity space provided at each school site for another physical activity space, yielding no net increase in overall minutes of physical activity. Fourth, because of the mixing of adult- and youth-centered programming at several of the school sites, observers in several instances could not determine with a strong degree of certainty whether the children observed participating in moderate to vigorous physical activity were part of a family unit (brought along by parents) or there for the youth-oriented activities. Finally, we observed community members using the school facilities at only 1 point in time. Therefore, whether these community members will continue to use the opened spaces for the longer term is difficult to predict.

In spite of analytic limitations, findings from the present study suggest that implementing school-based joint-use agreements is a promising strategy for increasing open space for moderate to vigorous physical activity among adults and children in under-resourced communities. Although Los Angeles County school districts have a history of creating such agreements for promoting physical activity among youth, they have not opened school facilities for use by adults. School officials have historically viewed children as the intended beneficiaries of school space. A new paradigm in which adults are welcomed to access school facilities for exercise is one step closer to the much-promoted model of schools as centers of the community (22,23). Agreements that stipulate physical activity programs for adults and youth, for example, may draw a much greater number of users from the community than agreements that do not.

Although our study highlights the potential of joint-use agreements for promoting physical activity in under-resourced communities, numerous questions remain. For example, what are the administrative and maintenance costs of these agreements for schools? How can local jurisdictions use joint-use agreements more effectively to engage community members in physical activity over longer periods of time, without significant attrition? What role does the family play in influencing community member participation at joint-use school sites? The latter question speaks to the interplay between adults and children as a family unit, which may offer social reinforcement for sustaining moderate to vigorous physical activity and can lead to multiplicative health benefits in the long term (17,18,22,23). Answers to these questions could help inform public health efforts to promote physical activity in disadvantaged communities.
